# Supplementary material for: Small RNA and Degradome Sequencing in Floral Bud Reveal Roles of miRNAs in Dormancy Release of Chimonanthus praecox
Source: Int J Mol Sci. 2023 Feb 20;24(4):4210. doi: 10.3390/ijms24044210 (PMC9964840; doi:10.3390/ijms24044210)
Supplement: Supplementary file 1 [file ijms-24-04210-s001.zip › Figure S2. Heatmap of 301 differentially expressed miRNAs (DEMs).pdf]

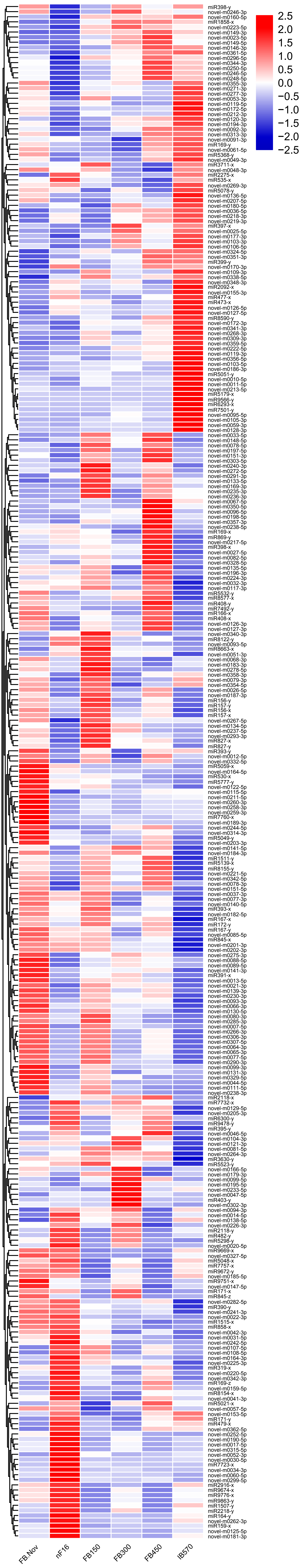
2.5
2.0
1.5
1.0
0.5
0.0
−0.5
−1.0
−1.5
−2.0
−2.5
FB.Nov
nF16
FB150
FB300
FB450
IB570
