# Supplementary material for: Small RNA and Degradome Sequencing in Floral Bud Reveal Roles of miRNAs in Dormancy Release of Chimonanthus praecox
Source: Int J Mol Sci. 2023 Feb 20;24(4):4210. doi: 10.3390/ijms24044210 (PMC9964840; doi:10.3390/ijms24044210)
Supplement: Supplementary file 1 [file ijms-24-04210-s001.zip › Supplementary Figures.pdf]

Supplementary Figures

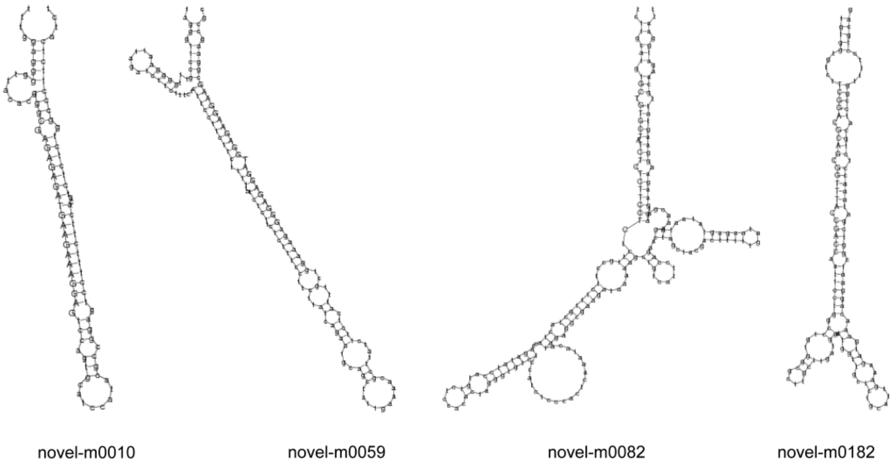

Figure S1. Predicted secondary structures of several novel miRNAs precursors. Mireap_v0.2 software with default parameters was used to prepare the hairpin structures of these novel miRNAs. Capital letters indicate mature miRNAs sequence.

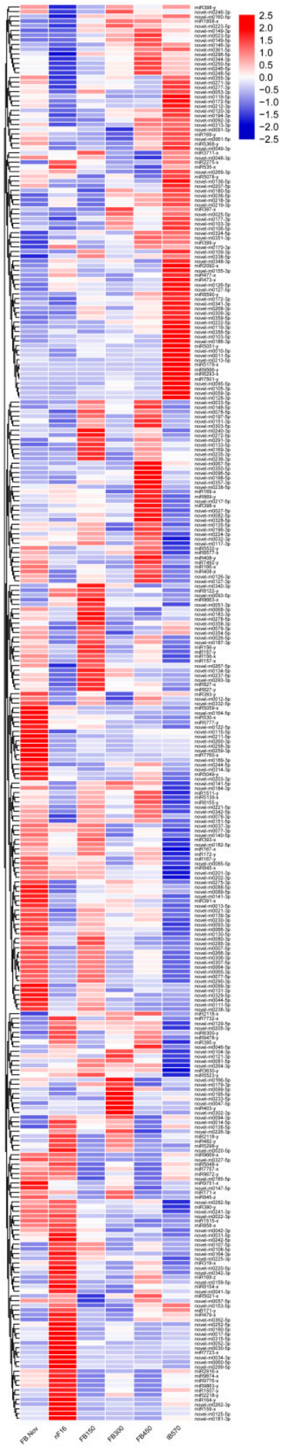

Figure S2. Heatmap of 301 differentially expressed miRNAs (DEMs) screened by comparisons in pairs among all 6 samples. Red represents up-regulation and blue represents down-regulation. It was completed by TBtools software with that the expression values of miRNAs were normalized using Z-score normalization.

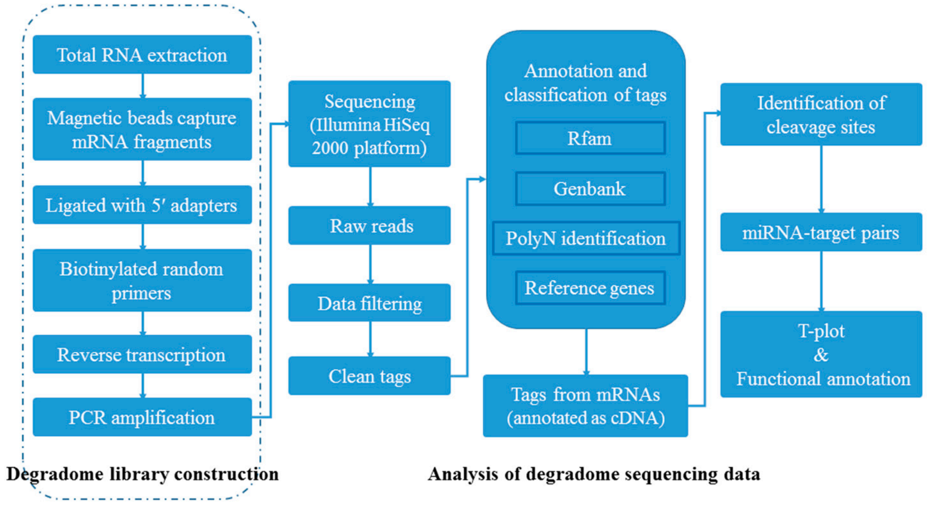

Figure S3. The process of the degradome sequencing
