# Supplementary figures and images for: Small RNA and Degradome Sequencing in Floral Bud Reveal Roles of miRNAs in Dormancy Release of Chimonanthus praecox
Source: Int J Mol Sci. 2023 Feb 20;24(4):4210. doi: 10.3390/ijms24044210 (PMC9964840; doi:10.3390/ijms24044210)

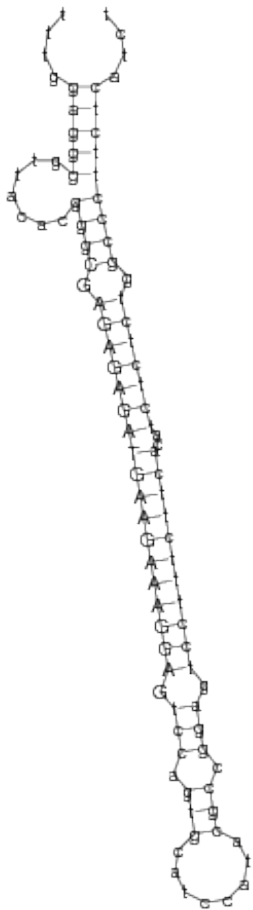

novel-m0010

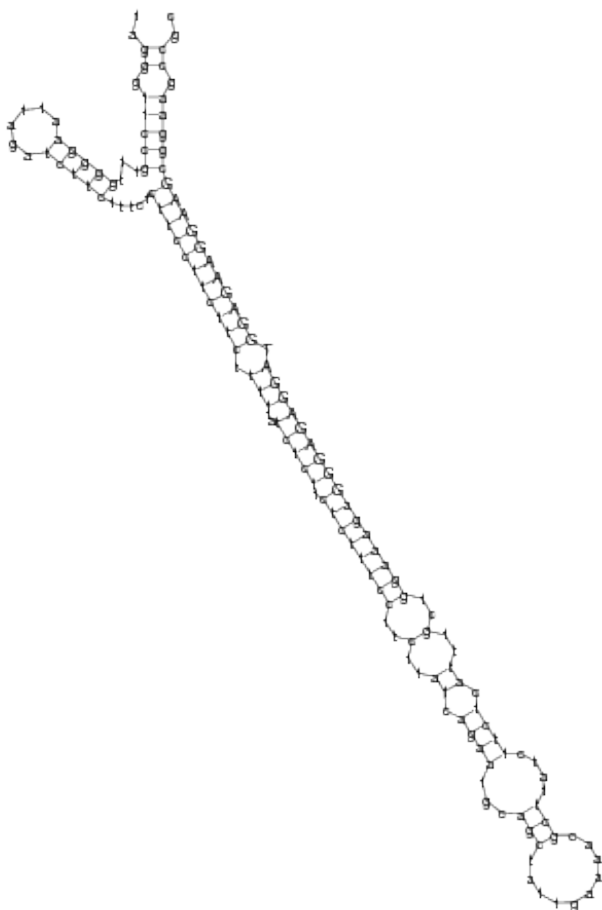

novel-m0059

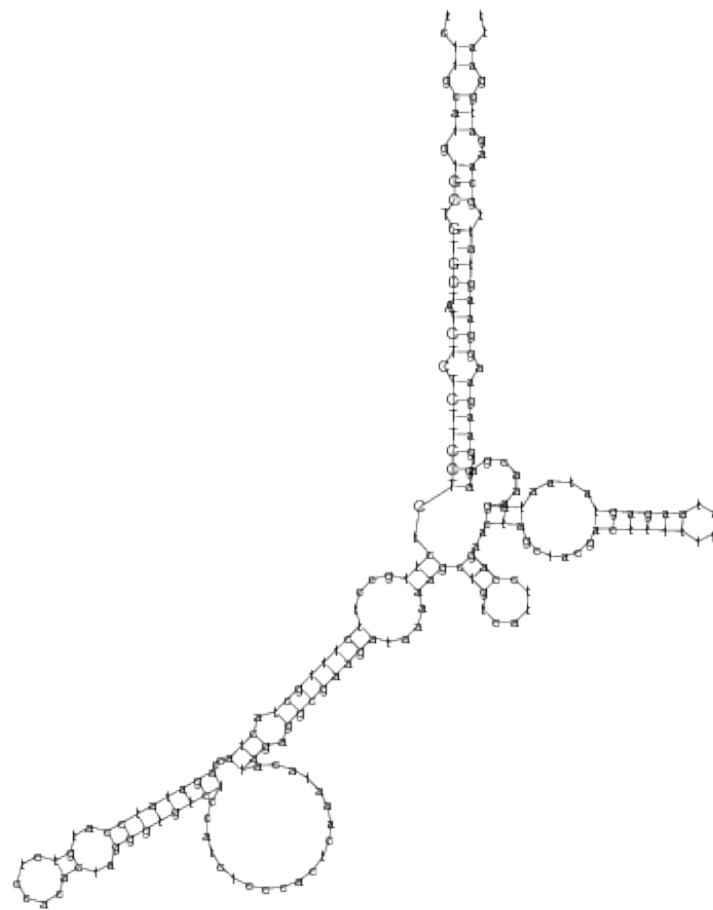

novel-m0082

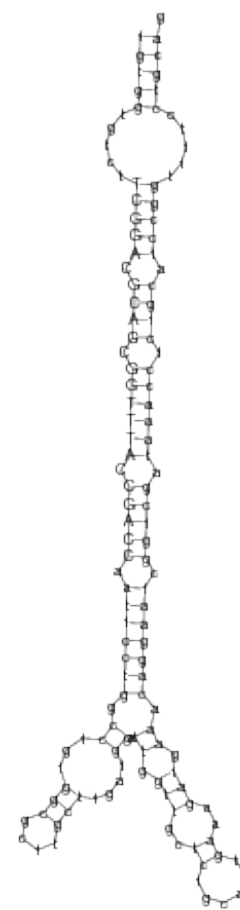

novel-m0182

Supplement: Supplementary file 1 [file ijms-24-04210-s001.zip › Figure S1. Predicted secondary structures of several novel miRNAs precursors.pdf]

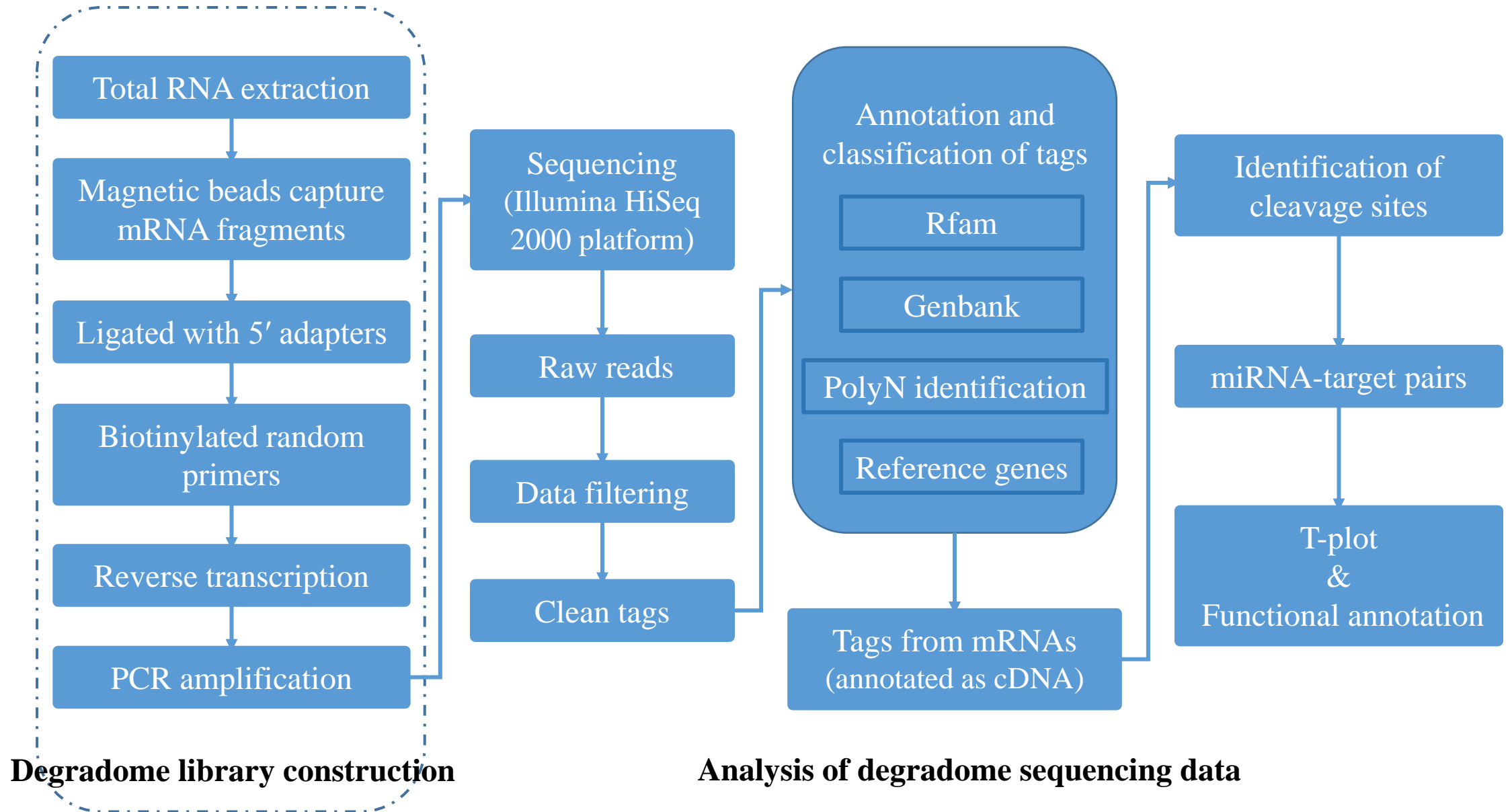

Supplement: Supplementary file 1 [file ijms-24-04210-s001.zip › Figure S3. The process of the degradome sequencing.pdf]

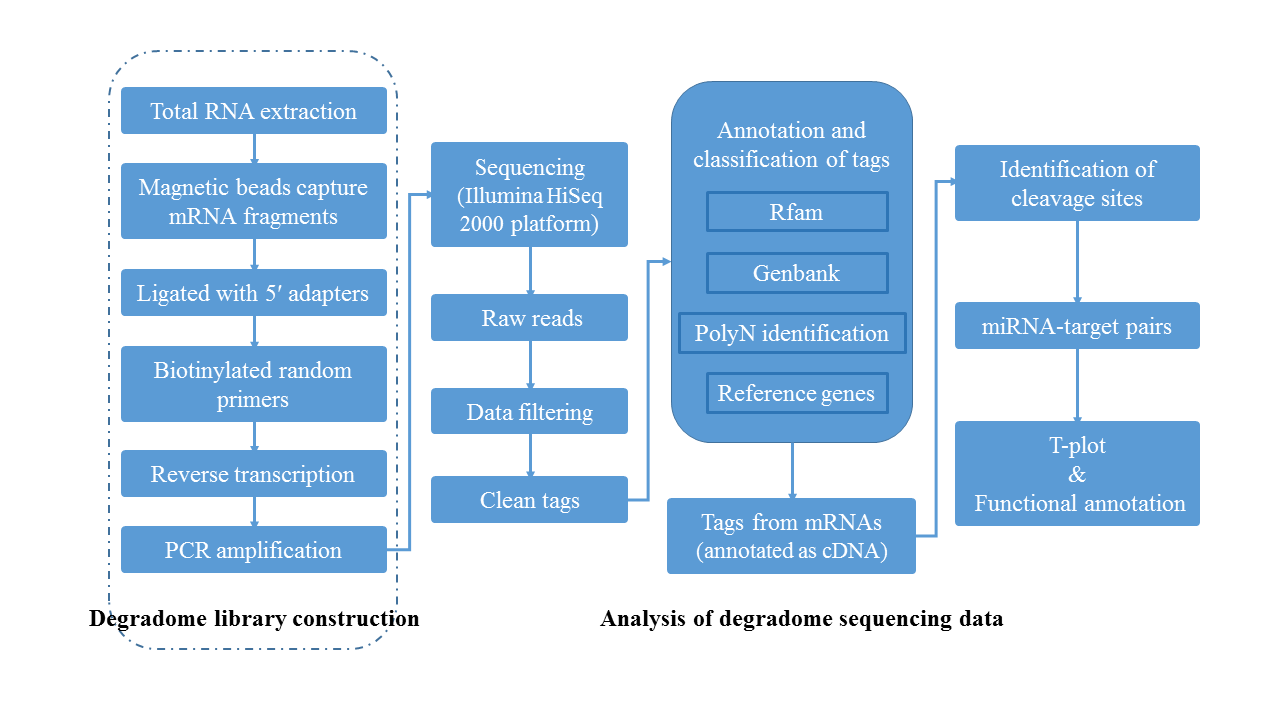

Supplement: Supplementary file 1 [file ijms-24-04210-s001.zip › Figure S3. The process of the degradome sequencing.tif]
